# Rosmarinic Acid Derivatives’ Inhibition of Glycogen Synthase Kinase-3β Is the Pharmacological Basis of Kangen-Karyu in Alzheimer’s Disease

**DOI:** 10.3390/molecules23112919

**Published:** 2018-11-08

**Authors:** Pradeep Paudel, Su Hui Seong, Yajuan Zhou, Chan Hum Park, Takako Yokozawa, Hyun Ah Jung, Jae Sue Choi

**Affiliations:** 1 Department of Food and Life Science, Pukyong National University, Busan 48513, Korea; phr.paudel@gmail.com (P.P.); seongsuhui@naver.com (S.H.S.); zhouyajuan26@gmail.com (Y.Z.); 2 Department of Medicinal Crop Research National Institute of Horticultural and Herbal Science Rural Development Administration, Eumseong 27709, Korea; ptman123@korea.kr; 3 Graduate School of Science and Engineering for Research University of Toyama, Toyama 930-8555, Japan; yokozawa@inm.u-toyama.ac.jp; 4 Department of Food Science and Human Nutrition, Chonbuk National University, Jeonju 54896, Korea

**Keywords:** Alzheimer’s disease, GSK-3β, Kangen-karyu, *Salvia miltiorrhiza*, salvianolic acids

## Abstract

Inhibition of glycogen synthase kinase 3β (GSK-3β) is considered to be the central therapeutic approach against Alzheimer’s disease (AD). In the present study, boiled water extracts of the Kangen-karyu (KK) herbal mixture and its constituents were screened for GSK-3β inhibitory activity. KK is used in traditional Kampo and Chinese medicines for improving cognitive function. The GSK-3β inhibition potential was evaluated by using the Kinase-Glo luminescent kinase assay platform. Furthermore, enzyme kinetics and in silico modeling were performed by using AutoDockTools to demonstrate the mechanism of enzyme inhibition. KK extract significantly inhibited GSK-3β in a concentration-dependent manner (IC_50_: 17.05 ± 1.14 μg/mL) when compared with the reference drug luteolin (IC_50_: 2.18 ± 0.13 μM). Among the six components of KK, extracts of Cyperi Rhizoma and Salviae Miltiorrhizae Radix significantly inhibited GSK-3β with IC_50_ values of 20.68 ± 2.50 and 7.77 ± 1.38 μg/mL, respectively. Among the constituents of the roots of *S. miltiorrhiza* water extract, rosmarinic acid, magnesium lithospermate B, salvianolic acid A, salvianolic acid B, and salvianolic acid C inhibited GSK-3β with IC_50_ values ranging from 6.97 to 135.5 μM. Salvianolic acid B was found to be an ATP-competitive inhibitor of GSK-3β and showed the lowest IC_50_ value (6.97 ± 0.96 µM). In silico modeling suggested a mechanism of action by which the hydrophobic, π–cation, and hydrophilic interactions of salvianolic acid B at ATP and substrate sites are critical for the observed GSK-3β inhibition. Therefore, one of the mechanisms of action of KK against AD may be the inhibition of GSK-3β and one of the active components of KK is the root of *S. miltiorrhiza* and its constituents: rosmarinic acid, magnesium lithospermate B, and salvianolic acids A, B, and C. Our results demonstrate the pharmacological basis for the use of KK against AD.

## 1. Introduction

Alzheimer’s disease (AD) is a progressive, irreversible neurodegenerative disease with a high rate of mortality among the elderly in developed countries. Even though the exact mechanism of AD pathogenesis is poorly understood, enzymes playing crucial roles in biochemical pathways involving AD development are known: viz. cholinesterase, β-amyloid cleaving enzyme 1 (BACE1), and glycogen synthase kinase 3 (GSK-3). The GSK-3 hypothesis of AD was recently proposed, according to which major hallmark characteristics of AD such as memory impairment, increased β-amyloid production, tau hyper-phosphorylation, and microglial-mediated inflammatory responses are the consequences of GSK-3 overactivity [1].

GSK-3 is a serine/threonine kinase that phosphorylates glycogen synthase and, thus, is linked to glycogen metabolism. Several studies by now have recognized it as a key component of a diverse array of cellular processes and diseases [2] including depression [3], anxiety [4], schizophrenia [5,6,7], Alzheimer’s disease [8,9], inflammation [10], various cancers [11,12], diabetes [13,14], and even AIDS [15]. Studies conducted to discover the linking bridge between GSK-3 and neuropathological features of Alzheimer’s disease have discovered the tau protein as a widely recognized substrate of GSK-3. GSK-3 is a prime candidate for contributing to the Alzheimer’s disease–associated hyperphosphorylation of tau [16]. However, increased production of amyloid-β (Aβ) peptides derived from the amyloid precursor protein (APP) by sequential proteolysis, as catalyzed by BACE1, has a controversial link to the GSK-3 enzyme. One study reported that, between two isomers of GSK-3 (GSK-3α and GSK-3β), GSK-3α rather than GSK-3β is involved in Aβ production [9]. Contrastingly, neither of the isomers of GSK-3 are involved in APP processing in vivo in the brain [17]. Nonetheless, many researchers believe that there is a strong correlation between the GSK-3 enzyme and APP processing [18] and amyloid-β-mediated tau phosphorylation [19]. In AD, GSK-3β is responsible for the phosphorylation of microtubule-associated tau protein, which affects microtubule stability and delocalization of the aberrant tau protein to brain cells and dendrites [20]. Following aggregation of hyperphosphorylated tau proteins, neurofibrillary tangles (NFTs) form, trigger synaptic dysfunction and neuronal death, which leads to cognition problems [21]. Since the active form of GSK-3β is at high levels in AD brains [22] and affects the stability of microtubules, which leads to neuronal cell death, some part of AD pathology could result from abnormal increase in GSK-3β. Therefore, secondary metabolites from natural sources that inhibit GSK-3β could be a promising strategy for AD treatment and, as recently reported, inhibition of GSK-3 can be achieved by N-terminal serine phosphorylation on GSK-3α (serine-21/S21) and GSK-3β (serine-9/S9), targets kinases such as protein kinase A and protein kinase B (PKB/AKT), and prevents GSK-3 binding to its substrates [23].

**Figure 1:**
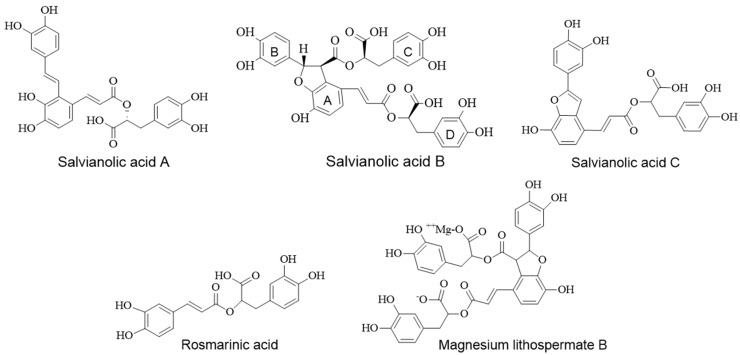
Structures of active polar components from *S. miltiorrhiza.*

Traditional Chinese and Japanese medicines have been a prime source of drug discovery in modern days. Among various traditional formulations, Kangen-karyu has gained much recent attention due to being a mixture of the six medicinal herbs Paeoniae Radix, Cnidii Rhizoma, Carthami Flos, Cyperi Rhizoma, Aucklandiae Radix, and Salviae Miltiorrhizae Radix. Kangen-karyu (KK) is a formula in traditional Kampo and Chinese medicine that has been used in treating neuro-degenerative diseases, diabetes, and various symptoms related to blood circulation. A handful of studies have reported the clinical relevance of using the KK extract such as for cognitive dysfunctions in type 2 diabetic patients by attenuating central cholinergic dysfunction [24], for age-related memory deficit by normalizing neuroplasticity-related signaling, and the VEGF system in the brain [25] against oxidative tissue damage in a mouse model [26], for diabetes and diabetic complications [27], for a neuroprotective effect [28], and for a renoprotective effect against diabetic nephropathy in type 2 diabetic mice [29]. However, there are no reports of GSK-3β inhibition by KK or its components. Thus, in this study, we evaluated the in vitro GSK-3β inhibition potential of water extracts of KK and its components. Additionally, polar compounds (Figure 1) from the active components were also evaluated by using simulation including simulation of enzyme kinetics and molecular docking.

## 2. Results

### 2.1. Glycogen Synthase Kinase-3β (GSK-3β) Inhibition

**Figure 2:**
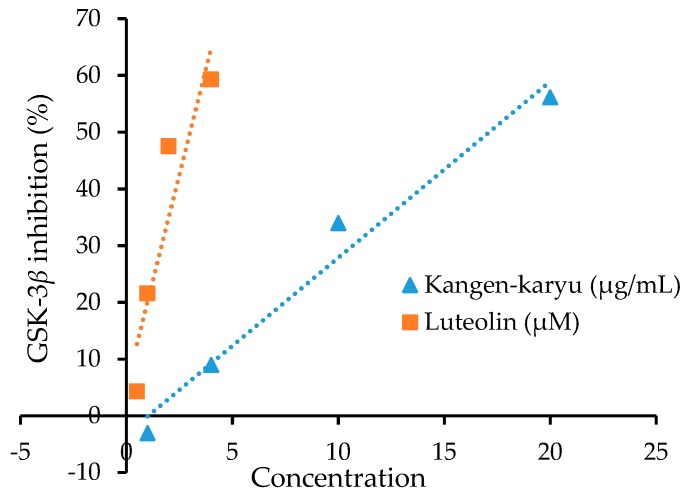
Linear graph of IC_50_ for glycogen synthase kinase-3β inhibition by water extract of Kangen-karyu and luteolin.

The water extract of KK and its individual components were evaluated for their inhibitory potential against GSK-3β in vitro. Figure 2 represents a linear graph of 50% inhibition of GSK-3β enzyme by KK and the reference compound luteolin. Table 1 tabulates the mean results for three different experiments on each component.

KK potently inhibited GSK-3β with the IC_50_ value of 17.05 ± 1.14 μg/mL. Similarly, all individual components displayed potent inhibition against GSK-3β with IC_50_ values ranging from 7.77 to 93.61 μg/mL. Salviae Miltiorrhizae Radix (IC_50_: 7.77 ± 1.38 μg/mL) was the most potent among them, which was followed by Cyperi Rhizoma (IC_50_: 20.68 ± 2.50 μg/mL). The other components displayed moderate to mild activity against GSK-3β.

Next, we evaluated the inhibitory potential of polar compounds (Figure 1) that are predominant in water extracts of *S. miltiorrhiza*. As listed in Table 2, the three salvianolic acids (Sal A, Sal B, and Sal C) and magnesium lithospermate B showed good inhibition against GSK-3β. Among them, Sal B was the most potent, which inhibited the enzyme by 50% at 6.97 ± 0.96 µM concentration. Sal A, Sal C, and magnesium lithospermate B each showed approximately one sixth the activity of Sal B with similar IC_50_ values of approximately 30 µM. In contrast, following the moderate activity of rosmarinic acid (IC_50_: 135.35 ± 4.69 µM), caffeic acid displayed mild inhibition (IC_50_: 425.01 ± 7.61 μM).

### 2.2. GSK-3β Enzyme Kinetics

**Figure 3:**
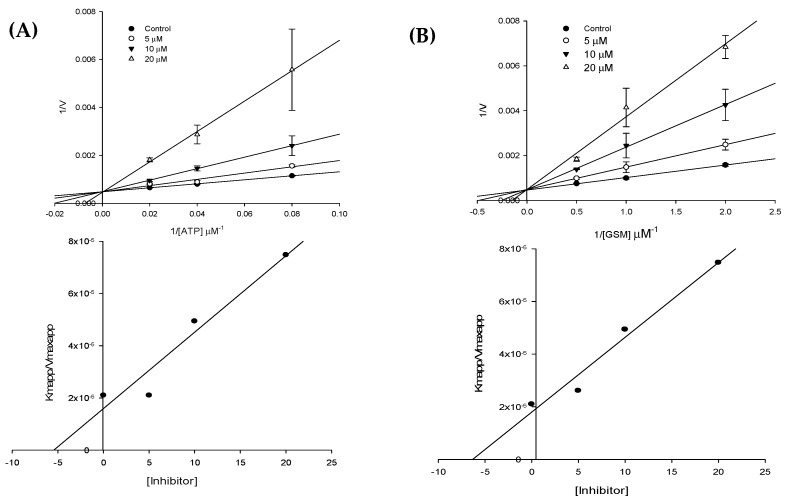
Lineweaver–Burk plots for GSK-3β inhibition by Sal B in the presence of various concentrations of (**A**) ATP and (**B**) GSM. Graphs below each Lineweaver-Burk plots represents secondary plots.

The mode of GSK-3β enzyme inhibition by Sal B was characterized via kinetic study by using various concentrations of GSM substrate and ATP. Lineweaver–Burk plots showing reciprocal rates versus reciprocal GSM substrate/ATP concentrations (Figure 3) were deployed to explore the GSK-3β inhibition mechanism. As shown in Figure 3, the double reciprocal plots yielded a group of lines with *y*-intercepts corresponding to the reciprocal of maximum velocity (*V*_max_) and slopes corresponding to the reciprocal of *V*_max_/*K*_m_. In both cases—(a) constant ATP (1 μM) with varying GSM substrate concentrations (12.5 μM, 25 μM, and 50 μM) and (b) constant GSM substrate (25 μM) with varying ATP concentrations (0.5 μM, 1 μM, and 2 μM)—the Lineweaver–Burk plot showed all lines intersecting at the same point on the *y*-axis. This suggested an unaltered 1/*V*_max_ and increase in *K*_m_ upon increasing the concentrations of Sal B. Table 2 lists the *K*_i_ values obtained from the secondary plots (Figure 3). Overall, the data indicated that Sal B competed with both the ATP and GSM substrates at their respective binding sites, which characterizes Sal B as an ATP-competitive and substrate-competitive inhibitor of GSK-3β.

### 2.3. Docking Studies for GSK-3β Inhibition

**Figure 4:**
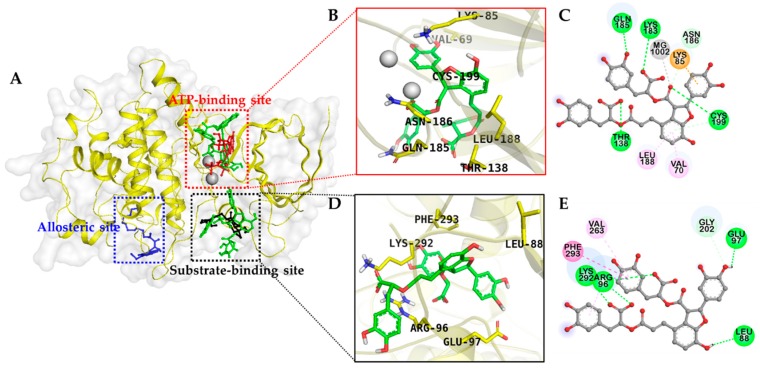
(**A**) Molecular docking of GSK-3β binding with salvianolic acid B and with positive controls. The chemical structures of phosphoaminophosphonic acid-adenylate ester (AMP-PNP), andrographolide, VP0.7, and salvianolic acid B are shown using red, black, blue, and green colored sticks, respectively. Magnesium ions are shown as gray spheres. Amino acid residues of enzyme are shown in yellow sticks. (**B**–**E**) Closer views of the salvianolic acid B binding pose in (**B**,**C**), the ATP binding site and (**D**,**E**) is the substrate binding site.

The binding pattern of Sal B on the active site cavity of GSK-3β was evaluated by using molecular docking studies employing AutoDock 4.2 (Figure 4). As shown in Table 3, Sal B formed H-bonds with GSK-3β residues Lys183 and Gln185 within the ATP binding pocket in a similar manner to the ATP-competitive reference compound, phosphoaminophosphonic acid–adenylate ester (AMP-PNP) and with Cys199 similar to the other ATP-competitive inhibitor, indirubin. Sal B formed an additional H-bond interaction with Thr138, which was not observed in either of the ATP-competitive reference inhibitors.

In addition, π–alkyl interactions with Leu188 and Val70 and a π–cation interaction with Lys85 were also observed with low binding energy (−6.18 kcal/mol), which demonstrates high binding affinity toward the ATP binding pocket (Figure 4).

**Figure 5:**
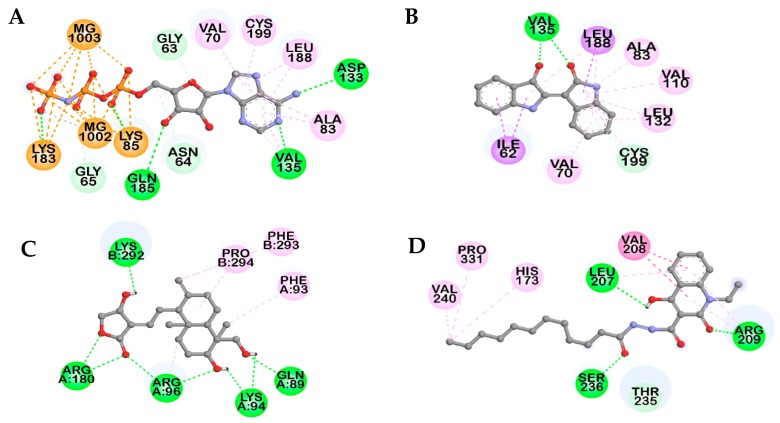
2D Diagram showing the binding of reference controls, AMP-PNP (**A**), indirubin (**B**), andrographolide (**C**), and VP0.7 (**D**), to GSK-3β active site.

Similarly, Sal B bound to the substrate binding pocket with −11.31 kcal/mol energy forms prime H-bond interactions with Arg96 and Lys292. Andrographolide used as a reference GSM substrate-competitive inhibitor to validate the docking result demonstrated for Arg96 and Lys292 as principal H-bond interacting residues (Figure 5). The other contributing interactions that occurred between Sal B and the substrate binding pocket were π–alkyl interactions with Arg96 and Val263, which is a π–π T-shaped interaction with Phe293, and a π–σ interaction with Lys292.

## 3. Discussion

With the reflection of pathological patterns, the approved ongoing treatment approaches for AD treatment are acetylcholinesterase inhibitors (AChEIs) (donepezil, galanthamine and rivastigmine, all raising acetylcholine levels) and uncompetitive *N*-methyl-d-aspartate (NMDA) receptor antagonist memantine, which normalizes dysfunctional glutamatergic neurotransmission [33]. However, these class drugs provide only symptomatic relief. Since memantine (glutamatergic) and AChEIs (cholinergic) target different pathological aspects of AD, combination therapy is rational for reducing the occurrence of marked clinical worsening in patients with moderate to severe AD, as compared to monotherapy [34]. Therefore, discovery of different targets that are crucial in AD pathogenesis is the prime focus of present days of which is a recently proposed GSK-3 hypothesis. GSK-3 plays a prime role in glucose metabolism and was initially identified as an enzyme that affects glycogen synthase (GS) phosphorylation. However, in recent years, it has been hypothesized that GSK-3 plays a more crucial role in the etiology of AD as a linking bridge between the amyloid-β and tau protein.

Increased activity of GSK-3 is responsible for tau hyperphosphorylation and neurofibrillary tangle formation. Therefore, GSK-3β represents a prime target for drug discovery for many awful and unmet human diseases.

Discovery of natural inhibitors that selectively target the substrate site of GSK-3β has materialized as a rational and realistic approach in AD drug discovery because ATP-site–directed GSK-3β inhibitors bind to many off-target kinases. Numerous chemically diverse groups of GSK-3 inhibitors have been discovered and periodically reviewed over the last decade. GSK-3β activity regulation is well studied in vitro and, in order for GSK-3β to be active, it requires phosphorylation at Tyr216 in the activation loop while it is inactivated by phosphorylation at the N-terminal Ser9 [35]. We conducted the present study with the aim to discover natural GSK-3β inhibitors and to put forth the basis of using KK in AD. Our results showed that KK dose-dependently inhibited GSK-3β enzyme. Since KK is a mixture of six medicinal herbs, its activity might not represent the activity of individual components and further discovery of inhibitor compounds from the herbal mixture would be quite tedious. This is why we also studied the individual components. Among the individual components, although most had moderate activity against GSK-3β, Salviae Miltiorrhizae Radix and Cyperi Rhizoma showed the greatest activity. Specific to GSK-3β inhibition, Salviae Miltiorrhizae Radix was twice as active as Cyperi Rhizoma and, thus, was selected for further study. In our previous study, regarding BACE1 inhibitors from *Salvia miltiorrhiza*, salvianolic acids A and C, rosmarinic acid, and magnesium lithospermate B, each of which are polar components, exhibited potent activity [36]. In this scenario, we evaluated the potentials of those polar compounds from the active water extract of *Salvia miltiorrhiza* against the GSK-3β enzyme.

Salvianolic acids are the main water-soluble component in *S. miltiorrhiza* among which Sal A and Sal B are the most abundant. Rosmarinic acid is a dimer, Sal A and Sal C are trimers, and Sal B and magnesium lithospermate B are tetramers of caffeic acid. These water soluble components had been reported as major components from the water soluble fraction of *Salvia miltiorrhiza* Bunge [37,38,39]. Review of the activities of our tested compounds showed that a monomeric form of caffeic acid displayed mild activity while its dimer rosmarinic acid showed a notable increment in activity. Trimers and tetramers exhibited prominent activity. This pattern reveals the insight that an increase in the number of caffeic acid moieties enhances activity. For kinetic study, Sal B was selected because it was the most active compound among those tested. We conducted the experiment using varying concentrations of ATP and GSM substrate individually. As discussed in our results section, Sal B was found to be both an ATP-competitive and GSM substrate–competitive inhibitor. In general, GSK-3β inhibitors are categorized as (a) ATP-competitive inhibitors, (b) non-ATP–competitive inhibitors, and (c) metal ion–competitive inhibitors (in the Mg^2+^ binding site). Phosphorylation of the key residues Arg96, Arg180, and Lys205 at the binding cavity regulate the activity of GSK-3β and Asp200, Glu97, and Lys85, which are important for ATP recognition and affinity because Asp200 interacts with the phosphate hydroxyl group of ATP [40]. In our docking study, the aromatic ring B of Sal B exhibited a π–cation interaction with the cationic Lys85 residue at the ATP recognition site. To some extent, this interaction might stabilize the orientation and binding conformation of the Sal B at the ATP binding site, which may enhance the binding affinity. The aromatic side chain of Phe or Tyr in the protein structure, usually involves π–cation interaction with the cationic side chain of Arg or Lys [41], and aromatic ligand binding to a protein with potential π–cation interaction contributes to a high binding affinity [21].

Various studies on salvianolic acids have demonstrated their neuroprotective effects. In a recent study, Sal B potently improved the proliferation and differentiation into neurons of induced pluripotent stem cells (iPSCs) via the AKT/GSK-3β signaling pathway [42]. Furthermore, Sal B inhibited A*β* generation by modulating BACE1 activity in SH-SY5Y-APPsw cells [43]. A study by Kim et al. [44] demonstrated that Sal B reverses cognitive impairments induced by scopolamine or A*β*_25–35_ via a GABAergic neurotransmitter system. Similarly, a review by Habtemariam [45] on the molecular pharmacology of rosmarinic acid and salvianolic acid in AD concluded that dimerization of caffeic acid or conjugation with another phenolic acid such as rosmarinic acid or a salvianolic acid might produce therapeutic agents for dementia. For any chemical to be developed as a drug, it must possess a desirable biological effect with optimum pharmacokinetic and pharmacodynamic properties. Investigation of the bioavailability, pharmacodynamics, and pharmacokinetics of salvianolic acids has demonstrated that Sal B reaches its maximum plasma concentration within 0.5–1 h due to first-order absorption and is also traceable up to 180 min after oral administration [46,47]. Evaluation of brain targeting of Sal B via nasal administration in rats has indicated higher brain-targeting efficacy (5.54) and bioavailability (43.98%) when compared to intravenous and oral administration [48]. A study on caffeic acid treated PC12 cells has revealed that caffeic acid at 20 µg/mL decreases the phosphorylation of GSK-3β and tau protein, which demonstrates a neuroprotective effect [49]. In our study, caffeic acid showed mild activity against GSK-3β inhibition in vitro. However, its dimer, rosmarinic acid, displayed potent activity. Since Sal B accounts for 57% of total phenolic acid content when compared to about 1% for Sal A [50] and Sal B was evidently a potent ATP-competitive inhibitor of GSK-3β in the present work, it might represent the prime component from salvia that could be developed into anti-AD drugs along with other rosmarinic acid derivatives.

Even though we conducted the present study on only one active component, it would be worthwhile to continue work on the other mildly active components because the activity of an extract does not represent the overall activity of its constituent compounds. Because the extract is a mixture of diverse groups of chemical components, its overall activity might vary from those of its individual components due to herbal–drug interactions having additive effects, synergistic effects, and/or antagonistic effects. A total extract showing high biological activity may contain inactive phytochemicals and an extract showing low activity might nonetheless contain active chemicals. Developing GSK-3β inhibitors is challenging due to some potential reports of toxicity such as hypoglycemia, tumorigenesis, and neuron deregulation [51]. Furthermore, since GSK-3 at a normal level is essential for normal physiology, its inhibition could destruct cellular functions. Therefore, a smooth inhibition of elevated GSK-3β to a physiological level is crucial and challenging. In addition, for GSK-3β inhibitors to be an effective drug for AD treatment, they should have specific brain distribution. The drug must cross the BBB to exert its action to regulate exacerbated GSK-3 brain levels. Hence, a further in vivo study must be conducted.

## 4. Materials and Methods

### 4.1. Plant Materials

Air-dried rhizomes of Paeoniae Radix, Cnidii Rhizoma, Carthami Flos, Cyperi Rhizoma, Aucklandiae Radix, and Salviae Miltiorrhizae Radix were purchased from a local retailer in the Daejeon city, Korea in January 2016. They were authenticated by Prof. J.S. Choi of Pukyong National University, Busan, Republic of Korea. A voucher specimen (No. 20160115) has been deposited in the laboratory of Prof. J.S. Choi.

### 4.2. Chemicals and Reagents

Test compounds were purchased from commercial sources. Caffeic acid and rosmarinic acid were obtained from Sigma-Aldrich (St. Louis, MO, USA). Salvianolic acids A and B (Sal A and Sal B) were purchased from TAUTO (Shanghai Tauto Biotech Co. Ltd., Shanghai, China) and Sal C was purchased from Herbest (Baoji Herbest Bio-Tech Co. Ltd., Baoji, China). All the tested compounds were of HPLC grade with ≥98% purity. Human recombinant GSK-3β was obtained from Prospec (ProSpec-Tany TechnoGene Ltd., Ness-Ziona, Israel). GSM substrate mimicking glycogen muscle synthase was obtained from Merck Millipore (Millipore Corporation, Temecula, CA, USA) and a Kinase-Glo kit was obtained from Promega (Promega Corporation, Madison, WI, USA). Adenosine 5-triphosphate (ATP) disodium salt hydrate, ammonium hydroxide, 4-(2-hydroxyethyl) piperazine-1-ethanesulfonic acid (HEPES), ammonium acetate, ethylene glycol-bis(-aminoethylether)-*N*,*N*,*N*′,*N*′-tetraacetic acid tetrasodium salt (EGTA), formic acid, ethylenediaminetetraacetic acid (EDTA), dimethyl sulfoxide (DMSO), 3-[(3-chloro-4-hydroxyphenyl)amino]-4-(2-nitrophenyl)-1H-pyrrol-2,5-dione, and magnesium acetate tetrahydrate were purchased from Sigma-Aldrich (St. Louis, MO, USA). All other chemicals and solvents were purchased from E. Merck, Fluka (Rupert-Mayer-Str., Munich, Germany) and Sigma-Aldrich unless otherwise stated.

### 4.3. Extract Preparation

The dried rhizomes were separately grinded to powder. Then the dried powder was gently boiled in water (with a water volume 25 times the powder volume) separately at 100 °C for 1 h. The total filtrate was concentrated to dryness in vacuo to yield water extracts (44% *w*/*w*) of each rhizome. Stock solutions of each extract were prepared in 100% DMSO.

### 4.4. GSK-3β Enzyme Inhibition Assay

The enzymatic inhibition of GSK-3β in vitro was evaluated with human recombinant GSK-3β by using a Kinase-Glo reagent kit, according to Baki et al. [52] with a slight modification. The kinase reaction was performed in 384-well black plates. Test concentrations were prepared through serial dilution by using an assay buffer (pH 7.5) containing 50 mM HEPES, 1 mM EDTA, 1 mM EGTA, and 15 mM magnesium acetate. To initiate the reaction, 5 µL of sample, 5 µL of ATP (1 µM final concentration), 5 µL of 50 µM GSM, and 5 µL of 20 ng GSK-3β were mixed simultaneously in each well (the final DMSO concentration in the reaction mixture was less than 5%). Test samples were replaced by 5 µL of either buffer or luteolin to produce positive control (maximum activity) and negative control (total inhibition) samples. The reaction mixture was incubated at 37 °C for 30 min and was followed by the addition of the 20-µL Kinase-Glo reagent to stop the reaction. Glow-type luminescence was recorded after 10 min. The activity was proportional to the difference between total and consumed ATP. Inhibition activities were calculated on the basis of maximal activity, measured in the absence of the inhibitor, and the maximal inhibition was measured in the presence of the reference compound.

### 4.5. GSK-3β Enzyme Kinetics

The GSK-3β enzyme kinetics was evaluated by using the assay method described in Section 4.4 with varying concentrations of ATP (2 µM, 1 µM, or 0.5 µM) or substrate (50 µM, 25 µM, or 12.5 µM). A Lineweaver–Burk plot was prepared by using double reciprocal plots of enzyme kinetic data. *K*_i_ values were calculated from the secondary plots.

### 4.6. Docking Studies for GSK-3β Inhibition

Molecular docking analysis was carried out using AutoDock 4.2 [53]. The X-ray crystallographic structure of human GSK-3β complexed with the known ATP-competitive inhibitor phosphoaminophosphonic acid–adenylate ester (AMP-PNP) was obtained at the resolution of 2.4 Å from the RCSB Protein Data Bank (PDB, ID: 1pyx) [54]. Rotatable bonds in the inhibitors and positive controls were assigned by AutoDockTools. The structures of salvianolic acid B and *N*′-dodecanoyl-1-ethyl-4-hydroxy-2-oxo-1,2-dihydroquinoline-3-carbohydrazide (VP0.7) were created using Chem3D Pro software (v12.0, CambridgeSoft Inc., Cambridge, MA, USA). The structures of indirubin (CID: 5359405) and andrographolide (CID: 5318517) was obtained from NCBI PubChem. Energy minimization of each ligand was carried out by using the Molecular Mechanics 2 (MM2) force fields method. AutoDock 4.2 was used for docking simulations and grid maps including ATP and substrate binding sites and allosteric sites were generated by using the AutoGrid program. The docking protocol for rigid and flexible ligand docking comprised 15 independent genetic algorithms. Docking results were visualized and analyzed by using PyMOL (v1.7.4, Schrödinger, LLC, Cambridge, MA, USA) and the Discovery Studio (v16.1, Accelrys, San Diego, CA, USA).

### 4.7. Statistical Analysis

All the results are presented as the mean ± standard error of the mean (SEM) of triplicate experiments. A one-way analysis of variance (ANOVA) and Duncan’s test (v23.0, Systat Inc., Evanston, IL, USA) were used to calculate the statistical significance. A *p*-value < 0.05 was considered significant.

## 5. Conclusions

We evaluated the anti-AD activity of KK and its individual components via GSK-3β. The results overall demonstrated that Salviae Miltiorrhizae Radix and Cyperi Rhizoma are the main active components of KK against GSK-3β. Furthermore, rosmarinic acid derivatives were discovered to be profound inhibitors. Among them, Sal B was a potent ATP-competitive inhibitor of the enzyme. Therefore, one of the mechanisms of action of KK against AD may be the inhibition of GSK-3β and one of the active components of KK is the root of *S. miltiorrhiza* and its constituents: rosmarinic acid, magnesium lithospermate B, and salvianolic acids A, B, and C. Our results demonstrate a pharmacological basis for the use of KK against AD.

## Figures and Tables

**Table 1:** Glycogen synthase kinase-3β inhibitory potentials of water extracts of Kangen-karyu and its constituents.

Samples	IC_50_ Values (Mean ± SEM) ^a^
Kangen-karyu	17.05 ± 1.14 ^f^
Aucklandiae Radix	85.04 ± 6.32 ^d^
Carthami Flos	93.61 ± 3.99 ^c^
Cnidii Rhizoma	66.74 ± 2.05 ^e^
Cyperi Rhizoma	20.68 ± 2.50 ^f^
Paeoniae Radix	62.51 ± 1.89 ^e^
Salviae Miltiorrhizae Radix	7.77 ± 1.38 ^g^
Luteolin ^b^	2.18 ± 0.13 ^h^

^a^ The 50% inhibitory concentrations (IC_50_; μg/mL) are expressed as mean ± SEM of triplicate experiments. ^b^ Used as a reference control (value in µM). ^c–h^ Mean with different letters are significantly different with Duncan’s test at *p* < 0.05.

**Table 2:** Glycogen synthase kinase 3β inhibitory potentials of polar compounds from water extract of *Salvia miltiorrhiza.*

Sample	IC_50_ (Mean ± SEM) ^a^	*K*_i_^c^	Inhibition Mode
Salvianolic acid A	30.21 ± 3.14 ^f^	-	-
Salvianolic acid B	6.97 ± 0.96 ^g^	5.44/6.32	Competitive
Salvianolic acid C	31.82 ± 2.08 ^f^	-	-
Caffeic acid	425.01 ± 7.61 ^d^	-	-
Rosmarinic acid	135.35 ± 4.69 ^e^	-	-
Magnesium lithospermate B	33.07 ± 3.88 ^f^	-	-
Luteolin ^b^	2.18 ± 0.13 ^g^	-	-
AR-A014418 ^b^	0.10	0.03	ATP-competitive [30]
Alsterpaullone ^b^	0.004	-	ATP-competitive [31]
Indirubin ^b^	0.60	-	ATP-competitive [32]

^a^ The 50% inhibitory concentrations (IC_50_, μM) are expressed as the mean ± SEM of triplicate experiments. ^b^ Used as a reference control. ^c^ *K*_i_ values for ATP-competitive and muscle glycogen synthase (GSM)-competitive inhibition, respectively. ^d–g^ Mean with different letters are significantly different with Duncan’s test at *p* < 0.05.

**Table 3:** Binding sites and docking scores of salvianolic acid B and reference inhibitors in GSK-3β.

Compounds	Binding Score (kcal/mol) ^a^	No. of H-Bonds	H-bonds Interacting Residues ^b^	Hydrophobic Interacting Residues ^b^	Others
AMP-PNP ^a^	−7.75 ^b^	11	Lys85, Val135, Lys183, Gln185, Asp133, Gly63, Asn64	π–Alkyl: Val70, Ala83, Leu188, Cys199, Ala83, Val135	Electrostatic bond: MG1002, MG1003, Lys85, Lys183
Indirubin ^a^	−7.67	3	Val135, Cys199	π-Alkyl: Leu188, Ala83, Val110, Leu132, Cys199, Val70, Leu132, Leu188, Pi-Sigma: Ile62, Leu188	
Andrographolide ^a^	−8.17	8	Gln89, Lys94, Arg96, Arg180, Lys292	Alkyl: Pro294, Arg96; π–Alkyl: Phe93, Phe293	
VP0.7 ^a^	−6.75	4	Arg209, Leu207, Ser236, Thr235	Alkyl: Val240, Pro331; π–Alkyl: His173, Arg209, Leu207; amide–π stacked: Val208, Arg209	
Salvianolic acid B	−6.18	4	Thr138, Lys183, Gln185, Cys199, Asn186	π–Alkyl: Leu188, Val70	π–Cation: Lys85; metal–acceptor: MG1002
	−11.31	7	Arg96, Lys292, Glu97, Leu88, Gly202	π–Alkyl: Arg96, Val263, π–π T-shaped: Phe293, π–σ: Lys292	

^a^ Phospho-aminophosphonic acid-adenylate ester (AMP-PNP) and indirubin are used as positive controls as ATP-competitive inhibitors. Andrographolide and *N*′-dodecanoyl-1-ethyl-4-hydroxy-2-oxo-1,2-dihydroquinoline-3-carbohydrazide (VP0.7) are used as positive controls and as substrate-competitive and allosteric inhibitor, respectively. ^b^ Root-mean-square deviation (RMSD) value: 0.98 Å.

## Abbreviations

AD	Alzheimer’s disease
ADT	AutoDockTools
GSK-3β	Glycogen synthase kinase-3β
KK	Kangen-karyu
TCM	Traditional Chinese medicine
